# Reversible Barrier Switching of ZnO/RuO_2_ Schottky Diodes

**DOI:** 10.3390/ma14102678

**Published:** 2021-05-20

**Authors:** Philipp Wendel, Dominik Dietz, Jonas Deuermeier, Andreas Klein

**Affiliations:** 1 Institute of Materials Science, Technical University of Darmstadt, 64287 Darmstadt, Germany; PhilippWendel@web.de (P.W.); dom.dietz@gmx.de (D.D.); 2 i3N/CENIMAT, Department of Materials Science, Faculty of Science and Technology, Campus de Caparica, Universidade NOVA de Lisboa and CEMOP/UNINOVA, 2829-516 Caparica, Portugal; j.deuermeier@fct.unl.pt

**Keywords:** Schottky barrier, resistive switching, zinc oxide, ruthenium oxide, oxygen vacancies

## Abstract

The current-voltage characteristics of ZnO/RuO${}_{2}$ Schottky diodes prepared by magnetron sputtering are shown to exhibit a reversible hysteresis behavior, which corresponds to a variation of the Schottky barrier height between 0.9 and 1.3 eV upon voltage cycling. The changes in the barrier height are attributed to trapping and de-trapping of electrons in oxygen vacancies.

## 1. Introduction

ZnO is a prototype wide band gap oxide semiconductor with a large defect tolerance [1]. Schottky diodes to the naturally n-type material have been studied extensively for applications such as photodetectors and field effect transistors or as mechanically tunable diodes in the field of piezotronics [2,3,4,5,6,7,8,9,10]. Mostly single crystalline or epitaxial thin films grown by pulsed laser deposition have been used in these studies. The prepared diodes exhibit remarkable properties, with rectification ratios up to $10^{10}$ and barrier heights up to 1.3 eV [6,8]. The highest barriers are usually obtained by materials having a low concentration of oxygen vacancies [3,11,12]. There are deep donor defects in ZnO, with an energy level of about 0.7–0.8 eV below the conduction band [13]. High concentrations will pin the Fermi energy and thereby determine the barrier height. The variation of oxygen related defects can even result in a conversion from ohmic to rectifying contacts, which has been demonstrated for ZnO/Au contacts [11]. For this, the concentration of bulk oxygen vacancies is not sufficient, as the deposition of metals onto oxide semiconductors typically results in a reduction in the surface induced by the heat of condensation of the metals, which results in a Fermi level pinning at the interface [14,15,16,17]. This reduction can be avoided by oxidizing deposition conditions or most directly by the deposition of conducting oxides [16,17]. Such strategies are also directly applied to achieve high Schottky barriers for electrons on ZnO or related oxide semiconductors [4,5,6,7,8,18,19].

With the described properties of ZnO Schottky diodes, in particular the influence of oxygen deficiency, a modification of barrier heights by chemical oxidation/reduction is possible. This variability of barrier height is much larger than that reported in dependence on strain but not convenient from the perspective of electronic applications due to expected slow switching times and likely elevated temperature requirements. In this work, a hysteretic current-voltage (IV) behaviour of ZnO Schottky barriers with an electrically programmable barrier height is reported. The Schottky barriers for electrons can reversibly be switched between 0.93 and 1.3 eV. Electrical control of resistance states in two-terminal devices is currently a potentially disruptive trend in information technology. The nonvolatile resistance states with analog distribution of memristors enable hardware neuromorphic computation systems that challenge von Neumann architectures, mainly in terms of efficiency [20,21]. Note, that training a software neural network for natural language processing running on a conventional computer architecture can produce five times as much carbon dioxide as a car during its life time [22].

Harvesting the ultimate circuit miniaturization potential of passive crossbar structures requires to solve the sneak path current issue, which is only rarely achieved with filamentary memristors [23,24,25]. An alternative is area-scaling devices with built-in rectification, which operate through barrier modulation. In the area-scaling mode, the set operation typically happens in the forward direction of the diode and allows the rectification to be maintained [26,27,28]. The saturation current is most effectively suppressed in the case of high barriers [29,30,31,32]. In this context, the devices presented here show promising characteristics, such as a substantial resistance window of at least four orders of magnitude (at 1.2 V), a gradual state distribution and a low saturation current, which is independent of the switching operation. Furthermore, the fabrication below 100 °C enables future implementations on plastic substrates. Hence, these devices may be interesting for low-voltage applications in flexible substrates, such as in wearables.

## 2. Materials and Methods

All thin film depositions were performed in the DArmstadt Integrated SYstem for MATerials research (DAISY-MAT), which combines several thin film deposition chambers with a Physical Electronics PHI 5700 surface analysis system via an ultra-high vacuum sample transfer system [17]. Thin films were prepared by magnetron sputtering using 2 inch diameter targets. Undoped and 2 wt.% Al-doped ZnO thin films were deposited from undoped and from Al-doped ceramic ZnO targets using 25 W radiofrequency (RF) excitation at 13.56 MHz, a 10 cm target-to-substrate distance, and a total gas flux of 10 sccm with a gas composition of either pure Ar or Ar/O${}_{2}$ mixtures. Both targets were installed in the same deposition chamber and were separated by apertures to avoid cross contamination. Deposition was performed with the substrate facing either target and selection of the target was carried out using a rotatable sample mount. Sample heating during deposition was performed using a halogen lamp located on the rotatable sample mount. Different substrate temperatures were tested. The best rectification and switching behavior was obtained using a substrate temperature of 100 °C.

In order to obtain reproducible rectifying diode characteristics, a systematic variation of the preparation conditions of the ZnO films including substrate temperature and oxygen content in process gas was performed. All diodes were prepared on glass substrates in out-of-plane geometry with an ohmic bottom contact and a rectifying top contact. Sn-doped In${}_{2}$O${}_{3}$ (ITO) and Al-doped ZnO (AZO) have been tried as bottom electrodes. Reproducible rectifying properties were only obtained with Al-doped ZnO. The poor diode characteristics with ITO electrodes were tentatively assigned to the higher mobility of oxygen in ITO [33], which may result in a stronger exchange of oxygen with the ZnO layer grown on top of the ITO layer. All diodes reported in this contribution have therefore been prepared on Al-doped ZnO bottom electrodes.

As the top contact material, we selected RuO${}_{2}$ which is known to form high barriers for electrons on oxide semiconductors. The advantage of using metal oxides instead of pure metals as contact material is that interface reactions, which lead to Fermi level pinning, can be avoided. For ZnO, Schottky barrier heights well above 1 eV have been determined using X-ray photoelectron spectroscopy (XPS) [16]. RuO${}_{2}$ films were deposited at room temperature through shadow masks with 50–200 µm diameter holes from a metallic Ru target with RF plasma excitation and a power of 25 W. A gas flux of 10 sccm with 7.5% O${}_{2}$ in Ar and a target-to-substrate distance of 10 cm were used. According to our measurements, the conductivity of RuO${}_{2}$ films grown with such conditions is ~$10^{4}\;S/\mathrm{cm}$. It is high enough to enable polarization reversal of ferroelectrics even at a thickness of only 2–3 nm [34].

In order to apply the shadow masks for top electrode deposition, the samples had to be removed from vacuum, which resulted in a contamination of the surface. Cleaning of the ZnO surface prior to RuO${}_{2}$ deposition was carried out by a remote oxygen plasma treatment, which had no significant effect on the electrical properties. On the other hand, heating in 0.5 Pa oxygen at 400 °C resulted in insulating contacts, which is also assigned to a reduction in the conductivity of the bottom contact.

Electric contacts to the bottom and top electrodes for current-voltage measurements were made by Au coated metal tips in a Signatone probe station using a home-made compartment for electrical shielding. Current-voltage curves were measured in the dark using a Keithley picoamperemeter controlled by a labview program. Measurements were performed with grounded bottom electrodes. The voltages were increased stepwise with 0.05 V increments. A delay time of 50 ms was applied before recording the current at each voltage step.

## 3. Results and Discussion

Diodes prepared on Al-doped ZnO bottom electrodes using a single ZnO layer grown at varying substrate temperatures and oxygen contents in the process gas indicated that films grown under more reducing conditions are too conductive and did not show any rectification, whereas films grown under more oxidizing conditions were highly resistive. The latter might be explained by the oxygen bombardment of the bottom electrode by negatively charged oxygen species in the plasma [35]. Such a bombardment should result in an oxygen excess in the ZnO:Al bottom electrode either by generating interstitial oxygen or Zn vacancies. These defects will compensate for the Al donors and thereby reduce the conductivity of the bottom electrode [36]. As a side effect, the lowered free electron concentration may also enhance grain boundary potential barriers and thereby reduce the carrier mobility [37].

**Figure 1:**
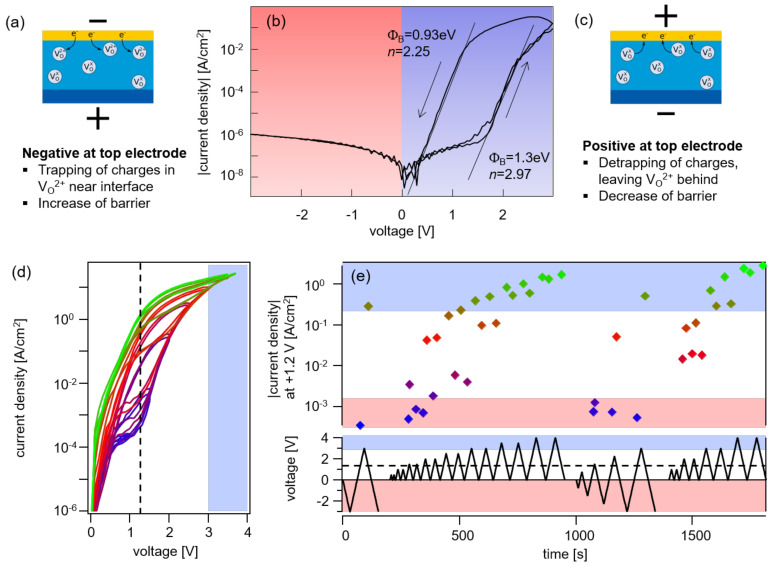
Current-voltage behaviour of AZO/ZnO:Ar/ZnO:O${}_{2}$/RuO${}_{2}$ layer structures. The sign of voltage refers to the polarity at the top contact. A typical IV curve obtained by cycling the voltage between −3 and +3 V is shown in (**b**). The indicated barrier heights, $\Phi_{B}$, and diode quality factors, *n*, were extracted from the 0 V intercept and the slope of the linear parts of the curves. The forward biased current obtained with a stepwise increasing voltage is shown in (**d**). The corresponding sequence of voltage for the curves shown in (**d**) and additional sequences are shown together with the extracted current densities at V=1.2 V (dashed lines) in (**e**). The proposed model to explain the switching behaviour is shown in (**a**,**c**).

Eventually, most reproducible diodes exhibiting good rectification were prepared using ZnO bilayers grown at 100 °C substrate temperature (see Figure 1b). The first layer, which is in contact with the bottom electrode, was grown under more reducing conditions in pure Ar (ZnO:Ar), with a thickness of ~20 nm, and the (thicker) second layer, which is in contact with the rectifying top contact, with 3% oxygen in the process gas (ZnO:O${}_{2}$). The total thickness of the nominally undoped ZnO bilayer was 300 nm.

A typical current-voltage curve of a highly rectifying AZO/ZnO:Ar/ZnO:O${}_{2}$/RuO${}_{2}$ diode is shown in Figure 1b. For small positive voltages, the current rises linearly up to ~1.5 V and then increases exponentially for higher voltages. Currents that are orders of magnitude higher were observed with a decreasing voltage. A blocking behaviour without hysteresis was observed for negative applied voltages. The observed behaviour was reversible when the voltage was varied between −3 and +3 V.

The hysteresis behaviour can be explained by a change in the Schottky barrier height upon voltage cycling. The low current for small positive voltages is likely related to a leakage of the diode, probably caused by surface conduction (no guard ring structure has been used for the top electrode). This leakage prevents lower currents and is superimposed on the diode current. The barrier height from the increasing current when increasing positive voltage (0→3 V) was extracted from the 0 V intercept of the linear part of the curve and amounts to 1.3 eV (see Figure 1b). This barrier is higher than most reported barrier heights for ZnO diodes [3,5] but similar to the one reported for oxidized Pt films [8]. Barrier heights of 1.3 eV for RuO${}_{2}$ and oxidized Pt are in good agreement with previous XPS measurements [16]. The barrier height extracted from the decreasing current for decreasing positive voltage (3→0 V) is only 0.93 eV. Apparently, the barrier height decreased at a high positive voltage and increased at a high negative voltage.

To further investigate this barrier switching behaviour, different sequences of voltage, indicated in Figure 1e, were applied to the diode structure. The IV curves recorded during a sequence of stepwise increasing maximum positive voltages are shown in Figure 1d. The curves indicate a stepwise decreasing barrier height with a gradual change in the hysteresis. The hysteresis disappears only after the voltage increased above a maximum of +3 V, for which the low barrier height was stabilized. The high barrier and the respective hysteresis were re-established once −3 V are applied to the diode. Apart from being a switchable diode, the prepared structures might also be used as memory device. The current density measured at V=+1.2 V can be switched by up to four orders of magnitude, as shown in the upper part of Figure 1e.

The change in barrier height cannot be explained by moving oxygen vacancies. They will only react to the electric field if they are charged. A negative applied voltage at the top electrode would then attract oxygen vacancies and increase the concentration of charged defects near the top electrode. This would narrow the space charge region and effectively lower the barrier, opposite to what is observed in the experiment. A model that can explain the observed changes in barrier height with voltage polarity is depicted in Figure 1a,c. It relies on the fact that oxygen vacancies are deep defects in ZnO [3,13]. These vacancies can trap electrons, which has been been assigned to the phenomenon of persistent photoconductivity [38]. For negative applied voltage, electrons are injected into ZnO. Once they are trapped in oxygen vacancies, they reduce the space charge density. Electrons are de-trapped for a positive applied voltage. This will increase the concentration of positively charged oxygen vacancies and consequently increase the electric field and thereby the image charge induced lowering of the effective barrier height, which is more pronounced in higher electric fields. Image force lowering also occurs in barrier switching devices based on the Pt/SrTiO${}_{3}$ Schottky contact, leading to analog memristive properties [39].

The reversible and persistent barrier height changes shown in this work are similar to charge-based memristors, which have been applied in passive cross-bar arrays [29]. As mentioned in the reference, the main benefit of these forming-free and self-rectifying devices is their much lower power consumption during programming, compared to memristors relying on ion migration. An essential requirement for the low power consumption during programming via an asymmetric voltage scheme is a high Schottky barrier, similar to the characteristics shown in Figure 1b, as a low saturation current density is maintained [29]. Another interesting application of memristors is as artificial synapse. Here, an analog distribution of resistance states is required [27]. Figure 1d,e show that a device with a less pronounced rectification can be easily tuned to intermediate states via the forward (or set) voltage range. Such a behaviour has also been observed for resistive switching devices with a Schottky contact between zinc-tin oxide (ZTO) and platinum [26], and was implemented in cross-point structures [28].

So far we do not have sufficient data to analyse the long term and temperature stability of the barrier switching mechanism. Being a charge trapping/de-trapping mechanism, the same limitations as in Flash technology apply [29]. As the barrier heights and trapping energies are several hundred meV, we do not expect major additional instabilities. The data in Figure 1 suggest that the switching occurs at ±3 V, but the device also withstands +4 V, indicating that a stable operation might be possible. Furthermore, degradation of oxides is often related to ionic motion [40]. A switching mechanism based on electronic charge carriers may be more stable.

Finally, the device performance known so far (and deposition temperature) shall be compared to relevant works from the literature, which are presented in Table 1:

The presented devices stand out from other works due to the combination of both a high rectification ratio and high resistance window. An additional advantage is a comparatively low fabrication temperature, which is crucial for applications on flexible substrates.

## 4. Conclusions

ZnO Schottky diodes with RuO${}_{2}$ contacts exhibit an electrically programmable barrier height with highest barriers of >1.3 eV. The variation of barrier height is assigned to trapping and de-trapping of electrons in oxygen vacancies. The barrier switching properties of the ZnO/RuO${}_{2}$ Schottky diodes show potential to be used as memristive devices. Excellent self-rectifying properties can be achieved in highly rectifying diodes, whereas an analog distribution of resistance states can be programmed in less rectifying devices. Further studies are required to judge whether this wide spectrum of barrier switching characteristics can be applied in hardware artificial intelligence systems.

## Figures and Tables

**Table 1:** A comparison of device characteristics with works from the literature (RT: room temperature).

Ref.	Year	Mechanism	Rectification	Window	Temperature (°C)
This work	2021	area-scaling	$10^{5}$	≥$10^{4}$	100
[32]	2021	area-scaling	$10^{4}$	$10^{4}$	250
[31]	2020	area-scaling	$10^{4}$	≥$10^{2}$	400
[30]	2019	area-scaling	>$10^{6}$	≈25	RT
[23]	2017	filamentary	>$10^{4}$	≈$10^{4}$	300
[29]	2016	area-scaling	>$10^{4}$	≈$10^{2}$	300

## Data Availability

The data presented in this study are available on request from the corresponding author.

## Abbreviations

The following abbreviations are used in this manuscript:

RF	Radiofrequency
ITO	Sn-doped In${}_{2}$O${}_{3}$
AZO	Al-doped ZnO
XPS	X-ray photoelectron spectroscopy
IV	Current-voltage
